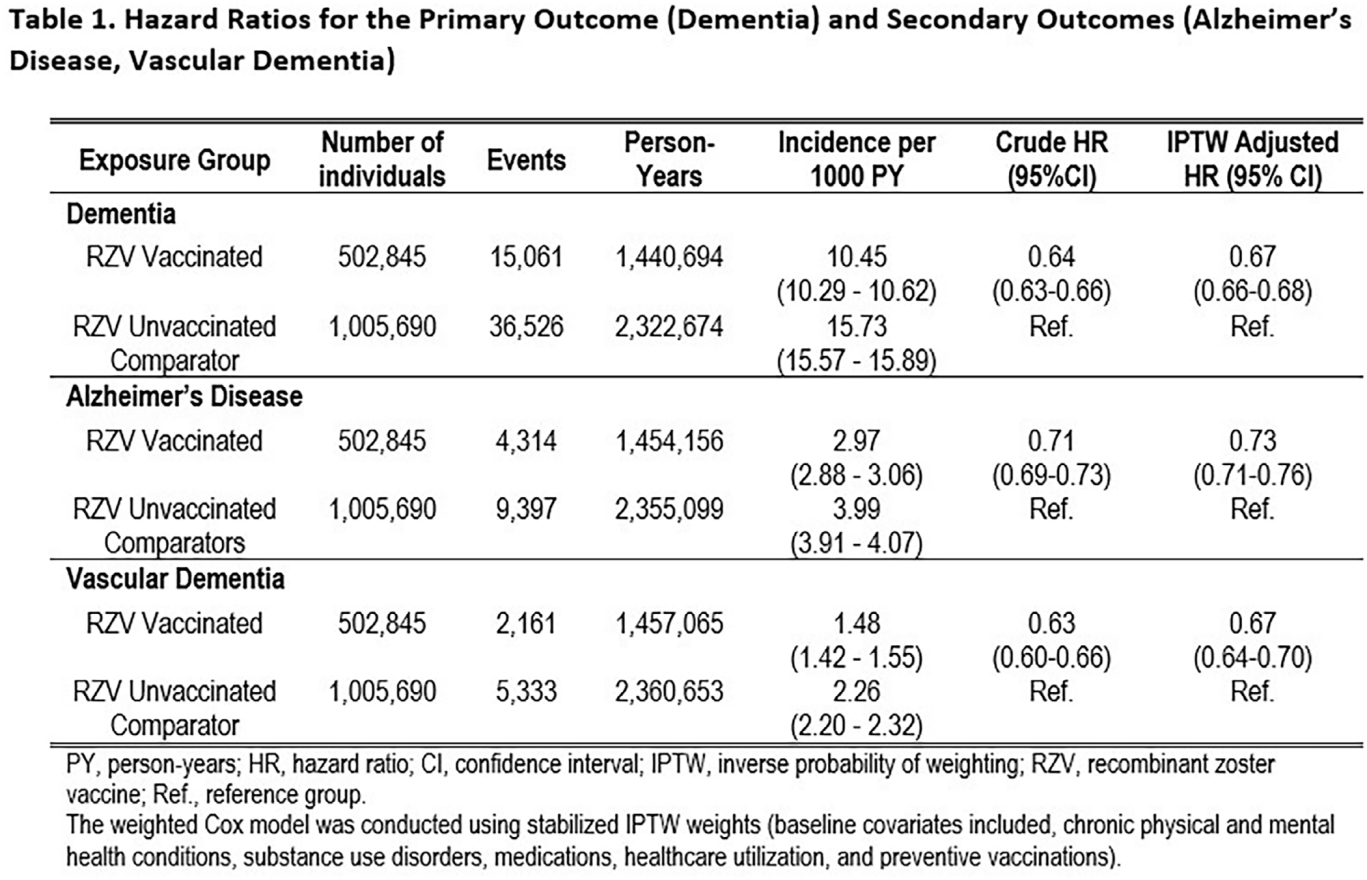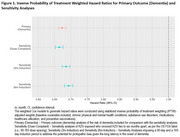# Recombinant Zoster Vaccine Associated with a Reduced Risk of Dementia Onset among US Beneficiaries ≥65 Years of Age

**DOI:** 10.1002/alz70861_108646

**Published:** 2025-12-23

**Authors:** Susan dosReis, Phuong Tran, Kareshma Mohanty, Alejandro Amill‐Rosario, Abree Johnson, Kathleen Ryan, Hannah Alsdurf, Driss Oraichi, Sofia B Pinto, Huifeng Yun

**Affiliations:** ^1^ University of Maryland, Baltimore, MD USA; ^2^ GSK, Rockville, MD USA; ^3^ GSK, Rio de Janeiro Brazil

## Abstract

**Background:**

Research showing lower risk of all‐cause dementia, Alzheimer’s Disease (AD), and vascular dementia (VD) with herpes zoster vaccination was based on one vaccine no longer marketed in the US. We evaluated the risk of dementia among US Medicare beneficiaries ≥65 years‐old who received recombinant zoster vaccine (RZV) relative to unvaccinated comparators.

**Methods:**

We implemented a matched cohort using Medicare fee‐for‐service claims from 2018‐2022 to identify individuals with two RZV doses (exposed). To mitigate healthy user bias, we selected individuals with a preventive care visit and no prior RZV (comparators). Each RZV exposed was matched to two comparators on 2‐year age bands, sex, and race/ethnicity in three‐month calendar intervals. Follow‐up started on day 1 post‐index date (RZV Dose 2; preventive care visit) and ended at the earliest of the outcome occurrence, loss of eligibility, death, receipt of *Zostavax* or *Shingrix* (for comparators), and 31/12/2022. The primary outcome was new‐onset dementia, and secondary outcomes were new‐onset AD and VD. Previously validated algorithms used ICD‐10 diagnosis codes in one inpatient or two outpatient encounters at least 7‐days apart to identify outcomes. Sensitivity analyses included 1) starting follow‐up at 3‐ and 6‐month post‐index date to address protopathic bias and 2) 2‐6‐month dose spacing per US label dose schedule. Cox proportional hazards models with stabilized inverse probability of treatment weights (IPTW) generated hazards ratios (HR) with 95% confidence intervals (CI).

**Results:**

The cohort of 502,845 RZV exposed, and 1,005,690 comparators was 92.83% non‐Hispanic White and 59.51% female, with a median age of 73 years. Incidence rates in the exposed and the comparators, respectively, were 10.45 versus 15.73 (dementia), 2.97 versus 3.99 (AD), and 1.48 versus 2.26 (VD) per 1000 person‐years. IPTW‐stabilized results in Table 1 show risk reductions of 33% (HR=0.67, 95% CI: 0.66‐0.68) for dementia, 27% (HR=0.73, 95% CI: 0.71‐076) for AD, and 33% (HR=0.67, 95% CI: 0.64‐0.70) for VD. Results from sensitivity analyses were similar to the primary analysis (Figure 1).

**Conclusions:**

There was a statistically significant reduction in the risk of new‐onset dementia, AD, and VD associated with a two‐dose RZV regimen.